# Childhood Symptoms of ADHD Overrule Comorbidity in Relation to Psychosocial Outcome at Age 15: A Longitudinal Study

**DOI:** 10.1371/journal.pone.0137475

**Published:** 2015-09-11

**Authors:** Eva Norén Selinus, Yasmina Molero, Paul Lichtenstein, Tomas Larson, Sebastian Lundström, Henrik Anckarsäter, Clara Hellner Gumpert

**Affiliations:** 1 Department of Clinical Neuroscience, Centre for Psychiatry Research & Education, Karolinska Institutet & Stockholm County Council, Stockholm, Sweden; 2 Department of Medical Epidemiology and Biostatistics, Karolinska Institutet, Stockholm, Sweden; 3 Center for Ethics, Law and Mental Health (CELAM), University of Gothenburg, Göteborg, Sweden; 4 Gillberg Neuropsychiatry Centre, University of Gothenburg, Göteborg, Sweden; National Center of Neurology and Psychiatry, JAPAN

## Abstract

**Objective:**

Neurodevelopmental problems (NDPs) may influence the transition from childhood to adolescence. Our aim was to study long-term psychosocial outcomes of NDPs, focusing on ADHD.

**Method:**

Data was collected through a telephone interview with parents of twins at ages 9 or 12 years. NDP screen-positive children were clinically assessed at age 15; N = 450. Psychosocial outcome concerning peers, school, internalizing problems, antisocial behavior, alcohol misuse, drug misuse, and impaired daily functioning was examined.

**Results:**

Even after controlling for other NDP comorbidity, screen-positivity for ADHD doubled or tripled the odds of later psychosocial problems. When controlling for parental education level, the significant effect of ADHD remained only for antisocial behavior and impaired daily functioning.

**Conclusions:**

Signs of NDPs as well as other psychiatric diagnoses at ages 9 or 12 years are associated with a more problematic adolescence. However, despite the presence of comorbidity, early ADHD symptoms stand out as the most important risk factor for later antisocial development and impaired daily functioning.

## Introduction

Childhood neurodevelopmental problems (NDPs; i.e., attention deficit and hyperactivity disorder [ADHD], autism spectrum disorder [ASD], tic disorder [TD], developmental coordination disorder [DCD], and learning disabilities [LD]), affect around 10% of all children [1]. ADHD is the most common disorder, the prevalence being between 5 and 10% [2–4], while the prevalence of ASD ranges between 1% and 2.6% [5–7]. About 1.5% of children meet criteria for LD [8–10], and 1% to 6.6% of children meet criteria for TD [11–14]. DCD has an estimated prevalence of about 5% [15, 16].

Children with NDPs have a higher risk of functional and psychosocial difficulties, increased mortality, and mental health problems, and often require a wide selection of life-long interventions from medical and social services [17]. Several studies have shown that children with ADHD are at risk for school failure, emotional difficulties, substance misuse, antisocial behavior and poor peer relationships in adolescence [18–23], and are more impaired in psychosocial, educational and neuropsychological functioning as adults [17]. Studies that have investigated psychosocial problems in NDPs other than ADHD are, however, scarce [24, 25]. The few studies that have examined associated problems of ASD, TD, LD, and DCD, have focused on psychiatric comorbidities [26, 27], academic difficulties [28, 29], and peer victimization [30, 31], and less attention has been paid to substance misuse and antisocial behavior [32–34]. Furthermore, research has been restricted by small samples, cross-sectional data or short-term follow-up [35, 36].

Children with one NDP diagnosis frequently demonstrate comorbidity with other NDP diagnoses [37–39]; for example, many children with ADHD also exhibit ASD symptoms [9, 40–43]. Studies have also reported large co-existence between TD and ADHD, LD and ASD [44–47]. Furthermore, up to 50% of children with LD or ADHD or ASD present DCD symptoms [48–50]. It has further been shown that individuals drift between NDP diagnoses [1, 51], leading to the suggestion that these diagnoses are not discrete disorders or syndromes. This notion was supported by findings from a study by Larson and colleagues [52]. In a Swedish sample screened for NDP diagnoses at ages 9 or 12 years (n = 198), about 50% received a clinical NDP diagnosis at 15, but only 40% of these received the same diagnosis as at the earlier screening. The same relationship was found in a Swedish clinical study [53].

NDP comorbidity is an important clinical feature and may have implications for diagnosis and treatment, as individuals with comorbid NPDs could present a more severe form of the disorders [32], and subsequently have a poorer long-term prognosis [51, 54]. Thus far, few studies have taken NDP comorbidity into account when examining long-term outcomes as most studies have examined outcomes of ADHD, ASD, DCD, LD and TD separately.

Given that NDPs are not entirely discrete disorders, comorbidity of NDPs needs to be systematically addressed both at baseline and follow-up in longitudinal studies, in order to examine if NDPs should be considered as a group of problem domains with shared outcomes. Likewise, it is equally important to consider a broad spectrum of psychosocial outcomes, since different symptom profiles may be linked to different outcomes.

### Aim

The aim of the present study was to examine the association between signs of ADHD and/or other NDPs (at ages 9 or 12 years) and negative psychosocial outcomes at age 15. Negative psychosocial outcome was defined as the presence of any of the following: peer problems, school/educational problems, internalizing problems, antisocial behavior, alcohol misuse, substance misuse, and/or impaired level of daily functioning/global impairment.

## Methods

### Subjects at Baseline

The Child and Adolescent Twin Study in Sweden (CATSS) [32] is an ongoing study that tracks all twins born in Sweden from July 1992 and onwards. A telephone interview including the **A**utism–**T**ics, **A**DHD, and other **C**omorbidities inventory (A-TAC) is conducted with the parents of all participating twins in connection with the children’s 9^th^or 12^th^ birthday (parents to twins born before July-95 were interviewed at age 12; those born July -95 and after were interviewed at age 9). As of January 2014, 12,500 A-TAC interviews had been performed, covering 25,000 twins. The overall response rate was 75%.

In the present study, 860 twins were eligible for inclusion. The response rate was 52%. 450 participated fully: 247 screen-positive for NDP and/or NDP related behavioral disorder (144 boys, 103 girls), 157 screen-negative (88 boys, 69 girls), and 46 randomly chosen sex-matched controls (30 boys, 16 girls). There were 38 twin pairs where both twins were screen-positive for a NDP (23 pairs of boys and 15 pairs of girls). An analysis of the attrition group in comparison with the study group is presented in Table 1.

**Table 1:** Descriptive statistics. Comparison of frequencies in the DOGSS cohort and the attrition cohort.

		DOGSS cohort (N = 450)	Attrition cohort (N = 410)	*P* ^A^
Sex	Girls	41.8%	31.7%	0.00
Screen-positives	Cases (NDP+non-NDP)	54.9%	58.5%	0.28
ADHD	ADHD screen-positive	21.1%	30.0%	0.00
Screen-negatives	Screen-negative cotwin	34.9%	32.7%	0.38
	Screen-negative control	10.2%	8.8%	0.47
Parental education level ^B^	Both parents have primary school or less (9 years of school)	3.1%	0.5%	0.01
	At least one of the parents has secondary school (10–12 years)	35.6%	41.2%	0.04
	At least one parent with university studies or equivalent	39.1%	35.1%	0.24

Note: Missing information on 22.2% of the DOGSS cohort, and 23.2% of the attrition cohort.

^A^ *P* value for comparison of distribution of qualitative variables between the DOGSS cohort and the attrition cohort with a Chi-square test

^B^ Parental education level: A combination of both parents’ education levels.

#### The A-TAC interview

The A-TAC was designed as an easy-to-administer, dimensional, and comprehensive interview to assess a broad scope of NDPs, and yields dimensional ratings of symptoms, suffering and dysfunction in a range of neurodevelopmental and psychiatric disorders, including ASD, ADHD, LD, TD and DCD. The investigators from a market research company (”Intervjubolaget”) who have all been trained in using the computerized version of the A-TAC inventory administer the telephone interview. All interviews are performed by laypersons. The average interview time for the A-TAC in the CATSS is about 30 minutes.

In previous validation studies, the A-TAC inventory has shown good test-retest measures, excellent inter-rater reliability and construct validity [52, 55–57], and convergent validity with the Child Behaviour Check List [58]. The Hansson et al study from 2005 showed excellent screening properties for ASDs and ADHD in a clinical sample [56]. Hansson et al found that Areas under Receiver Operating Characteristics curves between interview scores and clinical diagnoses were approx. 0.90 for ADHD and ASD and above 0.70 for TD, LD, and DCD. Using optimal cut-off scores for ASD and ADHD, good to excellent kappa levels for interviews and clinical diagnoses were noted. Larson and colleagues replicated these results in 2010 [57], and cut-off scores to identify proxies to clinical diagnoses were established for ASDs, ADHD, DCD, LD and TD. These cut-off scores (concurrent validity) had sensitivities above 0.90 (0.95 for ASD and ADHD). The predictive value for the A-TAC concerning the DOGSS cohort has also been addressed in Larson et al.’s study from 2013. The results in that study showed that the sensitivity and specificity of A-TAC scores for predicting later (3 or 6 years) clinical diagnoses were good to excellent, with values of the area under the receiver operating characteristics curves ranging from 0.77 (ADHD) to 0.91 (ASD).

### Screening at baseline (age 9/12)

In the A-TAC interview, cut-off scores for the different NDPs were determined and validated by two earlier studies [56, 57]. ADHD traits were calculated as the sum of scores of both the inattention and hyperactivity-impulsivity domains, yielding between 0 and 19 points. A dichotomous variable was created with a cut-off value of ≥8 points. The ASD cut-off level was set at ≥4.5 (of a maximum of 17 points), LD traits at ≥3.5 (out of 9), TD at ≥2 (out of 4) and DCD at ≥1.5 (out of 5) [57].

### Subjects at Follow-up

Twins born 1993–1995 (n = around 4100) were eligible for the clinical follow-up at age 15, referred to as the DOGSS study (Developmental Outcomes in a Genetic twin Study in Sweden). Same-sex twin pairs, in which at least one in the pair screened positive for one or several of the targeted disorders at ages 9/12 (i.e. ASD, ADHD, TD, LD, DCD, and/or behavioral disorders with known NDP comorbidities, such as Obsessive Compulsive Disorder [OCD] and/or Oppositional Defiant Disorder [ODD] and/or Conduct Disorder [CD] and/or Eating Disorder [ED]), were included. Randomly selected controls were included to create a population-based study group enriched for NDPs. From the 1995 cohort, the inclusion criteria were narrowed to include ASDs and ADHD only. The cohort (N = 860) was contacted by mail and invited to participate in a comprehensive clinical assessment, and a total of 452 adolescents consented to participate in the study (total response rate 52%). Two of the co-twins to screen-positive children had to be excluded from the present study due to missing A-TAC-data. In total, 450 participated fully in both CATSS 9/12 and DOGSS, including 247 screen-positive children (144 boys and 103 girls), 157 screen-negative co-twins (88 boys and 69 girls), and 46 randomly chosen, sex-matched controls (30 boys and 16 girls from CATSS-9/12).

### Procedure at follow-up

The follow-up assessment procedure included various self-report rating scales from a care-giver (i.e. the parent or parents that accompanied the twin to the clinical assessment participated) and the teenager, as well as a thorough clinical assessment. For the purpose of this paper, we used: a) data from the parent and adolescent self-reports regarding psychosocial functioning at age 15, and b) the clinical psychologists’ assessment of the teenager’s overall functional level (results from the diagnostic validation of the A-TAC interview have been reported elsewhere [52].

### Psychosocial outcome at age 15

Psychosocial outcome was defined as indicators of impairment in seven different domains related to everyday-life: peer problems, school problems, internalizing problems, antisocial behavior, alcohol misuse and/or drug misuse. In addition, a global measure of functioning, CGAS [59] was also used as an outcome variable.

#### Peer problems

**Peer problems** were assessed via the SDQ (Strengths and Difficulties Questionnaires) [60] using >5 points for self report (SR) and >3 points for parent report (PR) to indicate peer problems. The scale ranges between 0 and 10 points, where 0 is “no problems” and 10 is “serious problems”. The cutoff was chosen according to the standard for SDQ for abnormality. In addition, the inquiry of problems related to bullying was included. **Being bullied** was described using Olweus Bully Victim Questionnaire [61], a self-rating scale from 1 (not been bullied) to 5 (several times a week during the last months), and was dichotomized with the recommended cut-off value of ≥3. **Bullying others** was defined using the same questionnaire, with a scale from 1 (not bullied others) to 5 (several times a week during the last months), and was dichotomized with a cut-off value of ≥3. **Any Peer problem** was defined as a dichotomous variable, indicating that at least one of the scales described above reached above cut-off level.

#### School problems

**School problems** were defined as truancy, repetition of a school year and/or failure in grades. **Truancy** was assessed using Self-reported Delinquency [62–65], a self-rating scale, which was dichotomized with a cut-off value of ≥4 points (indicating more than 3–5 times of illegal absence from school). **Repetition of a school-year** was dichotomized as yes/no and **Failure in grades** was dichotomized as yes/no (Yes—failed in majority of subjects; No—passed in majority/all of subjects), as assessed in the K-SADS interview. **Any School problem** was defined as dichotomous (yes/no) variable indicating that at least one of the scales was scored above cut-off level.

#### Internalizing problems

**Internalizing problems** were assessed through self-rating and parent-rating scales from SDQ, including the sub-scores for Emotional symptoms. Each score´s range is between 0 (no problems) and 10 (serious problems). **Emotional symptoms** included psychosomatic symptoms, worry, unhappiness, nervousness, and fearfulness, and was dichotomized with a cut-off value of >6 for SR and >4 for PR, according to the standard for SDQ for abnormality. **Any internalizing problem** was defined as a dichotomous (yes/no) variable indicating that at least one of the scales was scored above cut-off level.

#### Antisocial behavior

**Antisocial behavior** was defined through a combination of three different self-reported measures of criminality, norm-breaking behavior and violent events using Self-reported Delinquency and SDQ. **Criminality** included frequency of self-reported: a) non-violent criminal acts and b) violent criminal acts. Non-violent criminal acts ranging from 0 to 10, was dichotomized with a cut-off value of ≥1 (at least one non-violent criminal act). Non-violent criminal acts were defined as vandalism, graffiti, fire, driving without a license, forgery of ID, car theft, theft, breaking into private property, and supplying illicit substances. Violent criminal acts was measured using self-reported violent behavior, including robbery, hurting animals, hurting a human, having sex with someone against her/his will with/without physical violence. It was expressed as frequency of self-reported violent acts, ranging from 0 to 6 and was dichotomized with a cut-off value of ≥1 (once or more of one or several acts). **Conduct problems** was assessed using: a) the K-SADS diagnose Conduct Disorder (yes/no) (which includes a Definite Diagnose [scored as 4 according to the K-SADS algorithm], a Probable Diagnose [3] and Diagnose in partial remission [5]) and b) SDQ Conduct Problems Score (self-rated and parent-rated). The SDQ questionnaire addressed conduct problems, and ranged between 0 (no problems) and 10 points (conduct problem several times a week during the last months). A dichotomous variable was created with a cut-off value of >4 points for SR and >3 points for PR, according to the standard for SDQ for abnormality. **Any Antisocial behavior** was defined as at least one of the antisocial problems above.

#### Alcohol misuse

**Alcohol misuse** was assessed using the Substance abuse self-reported alcohol and drug use [62–65] and included measures of drinking. **Drinking** was measured through two questions: a) “How often do you feel drunk when you drink alcohol?” ranging from 1 (“I don’t drink”) to 6 (“every time”) which was dichotomized with a cut-off value of ≥3 (rarely/sometimes/once in a while), b) “Have you been drinking beer, wine or liquor last month?” which was dichotomized into yes/no. **Any alcohol misuse** was defined as at least one of the alcohol problems above.

#### Drug misuse

**Drug misuse** was assessed using the Substance abuse self-reported alcohol and drug use [62–65], and included measures of drug use and substance abuse diagnosis (substance abuse diagnosis was assessed through the K-SADS interview). **Drug misuse** was defined as the reported use of any illicit drug and was dichotomized with a cut-off value of ≥1 (used at least one illicit drug). The K-SADS classifies the substance abuse diagnosis into a Definite Diagnose (4), a Probable Diagnose (3) and Diagnose in partial remission (5), and we included all of these. **Any drug misuse** was defined as at least one of the drug problems above.

#### CGAS

The Children’s Global Assessment Scale (CGAS) [59] is a clinician-rating tool used for both research purposes or in clinical settings. The rater is asked to assess the lowest overall level of psychosocial functioning during the preceding month. The scores range from 1 (the most impaired level) to 100 (the best level of functioning). CGAS is currently routinely used in many countries, and has also been used in several longitudinal and epidemiological studies [66–73]. Recent Swedish studies have been made concerning inter-rater reliability of CGAS [74] and comparing seminar and computer based training programs for CGAS on the accuracy and reliability of raters [75]. A third study showed that having a CGAS ≤ 60 was an independent predictor of several adversities in young adulthood, such as criminal conviction, bipolar disorder and borderline personality disorder [76]. **CGAS** was therefore dichotomized with a cut-off value of ≤ 60, which was then expected to identify adolescents at higher risk of developing different types of psychosocial problems. The participating psychologists were all trained in evaluation according to CGAS.

#### Parental education level

Data on the parents’ educational level was collected from the parent interview at age 9/12. It was divided into three levels: 1) Primary school (≤ 9 years of school) 2) Secondary school (10–12 years) 3) University studies or equivalent (> 12 years). This was recorded for both of the parents separately, but summarized in the analysis to a continuous variable ranging from 2 (both parents had level 1) to 6 (both parents had level 3).

### Statistical Analyses

First, frequencies for all seven negative psychosocial outcome domains, including sub-categories, are presented for the whole cohort (n = 450). In a second step, frequencies of all seven psychosocial outcomes are presented for each screen-positive diagnose at baseline (i.e. screen-positive according to the A-TAC interview; NDPs and other diagnoses), for screen-negative siblings of cases and for screen-negative random controls, respectively. Furthermore, a category was added to indicate the frequency of individuals with no reports in any of the seven negative psychosocial outcome domains, labeled as ‘No Problem’.

Since our definition of antisocial behaviors included a wide variety of antisocial acts, we also wanted to determine if the severity of antisocial behavior was associated differently to different NDP groups. We therefore distinguished between non-violent and violent antisocial behavior, as well as between the other sub groups included in the variable “Any antisocial behavior”, i.e. the SDQ conduct scale self- and parent-report, and CD diagnose. All of these behaviors are now displayed in a separate descriptive analysis.

Finally, we analyzed all cases (n = 293; i.e. all those individuals who were screen-positive for diagnoses at ages 9/12). Screen-negative co-twins were not included in this analysis since we wanted to compare screen-positives with pure controls and not with siblings (co-twins). For each individual we calculated the odds of each negative psychosocial outcome. The predictor of main interest in this analysis was ADHD, with or without comorbidity with other NDPs at ages 9/12. The association between ADHD and each of the outcomes was modeled with logistic regression, controlling for the following NDPs: co-existing autism spectrum disorder (ASD), learning disability (LD), tics disorder (TD), and developmental coordination disorder (DCD). The following non-NDPs were also controlled for: oppositional defiant disorder (ODD), conduct disorder (CD), obsessive compulsive disorder (OCD), and eating disorder (ED). In addition, we controlled for parental education level as described above. Since several correlated twin-pairs could be included in the analysis, a robust sandwich estimator was used to adjust for the correlated data when calculating the confidence intervals. The estimated associations are reported as odds ratios with 95% confidence intervals. All p-values less than 0.05 were considered statistically significant. The software package Stata IC12 was used.

### Ethical Considerations

For the CATSS study there was a written consent from the parent/legal guardian, and for the DOGSS study there was a written consent from both teenager and parent/legal guardian after written and oral information about the study. Analyses were performed on anonymized data files. The study protocol accorded with the Helsinki declaration and was approved by the ethical review board of Karolinska Institutet, Solna, Sweden (registration numbers CATSS Dnr: 02–289 and 2010/507-31/1 DOGSS Dnr: 03–672 and 2010/1356/31/1). Participants who were considered to be in need of child psychiatric care or social welfare, were taken care of in an adequate way.

## Results

The frequency rates of psychosocial outcomes are presented in Table 2. The most frequently reported negative outcome was ‘Any antisocial behavior’ (51.8%), followed by ‘Any alcohol misuse ‘(28.9%), ‘Any peer problem’ (23.6%), ‘Any school problem’ (23.6%), and ‘Any drug misuse’ (14.9%). ‘Impaired daily functioning’ was present in 15.3% of the participants (CGAS rate of ≤ 60; assessed by psychologist and child psychiatrist). Approximately 20% did not report any of the problems in focus.

**Table 2:** Distribution of age 15 psychosocial outcomes in the whole cohort (n = 450).

	n (%) of individuals	n (%) Missing ^A^
*Peer problems*		
Peer problems (SDQ) (SR)	46 (10.2)	7 (1.6)
Peer problems (SDQ) (PR)	67 (14.9)	7 (1.6)
Been bullied	12 (2.7)	32 (7.1)
Bullied others	12 (2.7)	26 (5.8)
Any Peer problem	106 (23.6)	0
*School problems*		
Truancy	78 (17.3)	15 (3.3)
Repeated a school-year	27 (6.0)	169 (37.6)
Failure (grades)	12 (2.7)	143 (31.8)
Any School problem	106 (23.6)	5 (1.1)
*Internalizing problems*		
Emotional problems (SDQ) (SR)	65 (14.4)	7 (1.6)
Emotional problems (SDQ) (PR)	43 (9.6)	7 (1.6)
Any internalizing problem	88 (19.6)	0
*Antisocial behavior*		
Non-violent Criminality	151 (33.6)	9 (2.0)
Violent act	133 (29.6)	9 (2.0)
Conduct problems (SDQ) (SR)	21 (4.7)	12 (2.7)
Conduct problems (SDQ) (PR)	44 (9.8)	58 (12.9)
Conduct Disorder diagnosis	10 (2.2)	0
Any Antisocial behavior	233 (51.8)	0
*Alcohol misuse*		
Intoxication	112 (24.9)	26 (5.8)
Used alcohol latest month	80 (17.78)	0
Any Alcohol misuse	130 (28.9)	0
*Drug misuse*		
Any Drug misuse	67 (14.9)	7 (1.6)
*CGAS ≤ 60*	69 (15.3)	4 (0.9)
*No problem* (of above)	89 (19.8)	0

Note: SR = self report; PR = parent report

^A^ Missing = Adolescents or parents who didn’t give a complete form of self reported information at age 15

Among the NDPs, antisocial behavior was the most common outcome across all proxy diagnoses (Table 3). Peer problems were also present across all the NDP proxy diagnoses (all above 30%, the ASD group reaching the highest level), but were also reported among the other groups. Those who had been screen-positive for ADHD displayed the highest rates of problems in the antisocial domain (66.3%), which was also the most frequent problem area for those who had been screen-positive for LD, TD and DCD. In addition to reporting the highest level of problems concerning peers, the ASD-group was assessed as having lower levels of functioning with 51.9% under the cut-off. School problems varied between 36.9% (ADHD group) and 17.1% (TD group). Internalizing problem levels were all in the 20%-range, with the highest for the ASD group (29.6%). Alcohol misuse was most common in the ADHD group, and the DCD group reported the highest frequency of drug misuse. Those who had been screen-positive for other diagnoses (ODD, CD, OCD or ED) also reported higher frequencies of all negative outcomes, as compared with screen-negative siblings and controls. Antisocial behavior and other problems were the most common outcome in CD/ODD (over 70%), but the cases in this sample were few which limits the generalizability. Screen-positive individuals all displayed more impaired levels of functioning as compared with screen-negative siblings or controls. Screen-negative siblings of cases showed lower rates of problems than the NDP cases, but higher than the screen-negative controls.

**Table 3:** Screen-positive cases according to A-TAC, screen-negative siblings, and random, screen-negative controls compared to the psychosocial outcomes at age 15. ***Baseline***: CATSS-9/12 study (n = screen-positive in A-TAC for “proxy” diagnosis). ***Follow-up***: Psychosocial outcomes in the clinical follow-up study, CATSS-15/DOGSS.

	Psychosocial outcomes at age 15							
“Proxy” diagnoses at Baseline age 9/12	Any peer problem n (%)	Any school problem n (%)	Any interna-lizing problem n (%)	Any anti-social behavior n (%)	Any Alcohol misuse n (%)	Any Drug misuse n (%)	CGAS (≤ 60) n (%)	No problem n (%)
*Screen-positive for ADHD* ^*A*^ *n = 95*	32 (33.7)	35 (36.9)	20 (21.1)	63 (66.3)	38 (40.0)	19 (20.0)	33 (34.7)	12 (12.6)
*Screen-positive for ASD* ^*A*^ *n = 27*	18 (66.7)	5 (18.5)	8 (29.6)	11 (40.7)	2 (7.4)	5 (18.5)	14 (51.9)	2 (7.4)
*Screen-positive for LD* ^*A*^ *n = 74*	25 (33.8)	22 (29.7)	15 (20.3)	32 (43.2)	23 (31.1)	12 (16.2)	26 (35.1)	15 (20.3)
*Screen-positive for TD* ^*A*^ *n = 35*	12 (34.3)	6 (17.1)	7 (20.0)	20 (57.1)	10 (28.6)	6 (17.1)	10 (28.6)	3 (8.6)
*Screen- positive for DCD* ^*A*^ *n = 38*	14 (36.8)	8 (21.1)	9 (23.7)	15 (39.5)	10 (26.3)	11 (29.0)	7 (18.4)	8 (21.1)
*Screen- positive for ODD* ^*B*^ *n = 47*	18 (38.3)	22 (46.8)	11 (23.4)	37 (78.7)	19 (40.4)	8 (17.0)	15 (31.9)	3 (6.4)
*Screen- positive for CD* ^*B*^ *n = 4*	1 (25.0)	2 (50.0)	1 (25.0)	3 (75.0)	3 (75.0)	1 (25.0)	1 (25.0)	0 (0)
*Screen- positive for OCD* ^*B*^ *n = 38*	17 (44.7)	6 (15.8)	10 (26.3)	19 (50.0)	8 (21.1)	5 (13.2)	11 (29.0)	7 (18.4)
*Screen- positive for ED* ^*B*^ *n = 13*	5 (38.5)	4 (30.8)	2 (15.4)	7 (53.9)	3 (23.1)	1 (7.7)	5 (38.5)	3 (23.1)
*Screen-negative siblings of cases n = 157*	26 (16.6)	30 (19.1)	29 (18.5)	86 (54.8)	42 (26.8)	19 (12.1)	6 (3.8)	30 (19.1)
*Screen-negative random controls n = 46*	5 (10.9)	3 (6.5)	5 (10.9)	16 (34.8)	10 (21.7)	6 (13.0)	2 (4.4)	16 (34.8)
*N = 450*	106 (23.6)	106 (23.6)	88 (19.6)	233 (51.8)	130 (28.9)	67 (14.9)	69 (15.3)	89 (19.8)

^A.^ **NDP** defined as ASD and/or ADHD and/or LD and/or TD and/or DCD, with a possible overlap of other mental health problems. NDP screen-positive: n = 198.

^B.^ **Other mental health problems** defined as OCD and/or ODD and/or CD and/or ED, with no NDP overlap. Screen-positive for other mental health problems: n = 49.

An analysis of sub categories of ‘Any antisocial behavior’ was done to determine the severity of antisocial behavior in the different groups (Table 4). In the ADHD group, 41.1% reported non-violent criminality, 31.6% reported violent acts, and 26.3% of their parents reported conduct problems in SDQ. Also, the rate of CD diagnosis was highest in the ADHD group: 7.4%. The ASD, LD, and DCD groups were lower in all sub categories, whereas the TD group reported both higher non-violent criminality (45.7%) and violent acts (40.0%). Of the Non-NDP groups, the ODD group surpassed the ADHD group in all sub categories except CD diagnosis (6.4%). Screen-negative siblings reported higher frequencies in both non-violent criminality (36.9%) and violent acts (34.4%) than did controls (15.2% and 23.9%). In general, non-violent criminality was more common than violent acts.

**Table 4:** Frequencies of subcategories of the outcome antisocial behavior (see Table 3, column 4) for screen-positive cases, screen-negative siblings, screen-negative controls, and the whole cohort at age 15.

	Antisocial behavior at age 15				
“Proxy” diagnoses at Baseline age 9/12	SDQ Conduct* (SR) n (%)	SDQ Conduct* (PR) n (%)	Non-violent criminality (SR) n (%)	Violent act (SR) n (%)	CD diagnosis (K-SADS) n (%)
*Screen-positive for ADHD* ^*A*^ *n = 95*	6 (6.3)	25 (26.3)	39 (41.1)	30 (31.6)	7 (7.4)
*Screen-positive for ASD* ^*A*^ *n = 27*	1 (3.7)	5 (18.5)	6 (22.2)	3 (11.1)	0
*Screen-positive for LD* ^*A*^ *n = 74*	4 (5.4)	5 (6.8)	22 (29.7)	13 (17.6)	3 (4.1)
*Screen-positive for TD* ^*A*^ *n = 35*	1 (2.9)	3 (8.6)	16 (45.7)	14 (40.0)	0
*Screen- positive for DCD* ^*A*^ *n = 38*	0	5 (13.2)	9 (23.7)	8 (21.1)	1 (2.6)
*Screen- positive for ODD* ^*B*^ *n = 47*	5 (10.6)	16 (34.0)	23 (48.9)	18 (38.3)	3 (6.4)
*Screen- positive for CD* ^*B*^ *n = 4*	0	3 (75.0)	1 (25.0)	2 (50.0)	0
*Screen- positive for OCD* ^*B*^ *n = 38*	3 (7.9)	7 (18.4)	9 (23.7)	9 (23.7)	1 (2.6)
*Screen- positive for ED* ^*B*^ *n = 13*	1 (7.7)	1 (7.7)	5 (38.5)	5 (38.5)	0
*Screen-negative siblings of cases n = 157*	7 (4.5)	8 (5.1)	58 (36.9)	54 (34.4)	0
*Screen-negative random controls n = 46*	2 (4.4)	0	7 (15.2)	11 (23.9)	0
*N = 450*	21 (4.7)	44 (9.8)	151 (33.6)	133 (29.6)	10 (2.2)

**Baseline**: CATSS-9/12 study (N = screen-positive in A-TAC for “proxy” diagnosis); **Follow-up**: Five subcategories of Antisocial behavior in the clinical follow-up study, CATSS-15/DOGSS.

^A.^ **NDP** defined as ASD and/or ADHD and/or LD and/or TD and/or DCD, with a possible overlap of other mental health problems. NDP screen-positive: n = 198.

^B.^ **Other mental health problems** defined as OCD and/or ODD and/or CD and/or ED, with no NDP overlap. Screen-positive for other mental health problems: n = 49.

* Questions included in the **SDQ Conduct Problems Scale**: 1. Often has temper tantrums or hot tempers; 2. Generally obedient, usually does what told (reversed question); 3. Often fights with other children or bullies them; 4. Often lies or cheats; 5. Steals from home, school or elsewhere

**SR** = self report; **PR** = parent report

Table 5 shows an in-depth analysis through logistic regression of the NDP subsample with signs of ADHD at ages 9 or 12 years in relation to the other groups. Having been screen-positive for ADHD at 9/12 doubled the odds for school and antisocial problems at age 15 (‘Any school problem’ OR 2.28, 95% CI 1.30–4.01; ‘Any antisocial behavior’ OR 2.67, 95% CI 1.58–4.52), and increased the odds of risky alcohol use (OR 1.97, 95% CI 1.10–3.53). Adjusting for ASD, LD, TD, DCD, and also for ODD, CD, OCD and ED, did not change the odds materially. However, when adjusting for parental education level, the significant association disappeared both for school problems and risky alcohol use, but remained for antisocial behavior (OR 2.16, 95% CI 1.14–4.09) at a slightly lower level. The significant association between ADHD and functional level remained (and even increased) in the adjusted model.

**Table 5:** Logistic Regression Analyses of the Associations of ADHD at Age 9/12 and Seven Psychosocial Outcomes at Age 15.

Baseline A-TAC in CATSS 9/12 “Proxy” diagnoses	Any peer problem OR (CI)	Any school problem OR (CI)	Any internalizing problem OR (CI)	Any antisocial behavior OR (CI)	Any alcohol misuse OR (CI)	Any drug misuse OR (CI)	CGAS (≤60) OR (CI)
ADHD (n = 95)	1.59 (0.90,2.80)	2.28** (1.30,4.01)	1.09 (0.59,1.99)	2.67*** (1.58,4.52)	1.97* (1.10,3.53)	1.48 (0.79,2.76)	3.13*** (1.67,5.87)
ADHD adjusted for ASD	1.33 (0.73,2.44)	2.39** (1.34,4.26)	1.02 (0.55,1.90)	2.91*** (1.68,5.03)	2.32** (1.28,4.21)	1.47 (0.78,2.77)	2.81** (1.48,5.35)
ADHD adjusted for LD	1.63 (0.92,2.89)	2.32** (1.32,4.07)	1.09 (0.59,1.99)	2.64*** (1.56,4.49)	1.98* (1.11,3.55)	1.48 (0.79,2.78)	3.66*** (1.91,7.02)
ADHD adjusted for TD	1.59 (0.90,2.82)	2.29** (1.30,4.03)	1.09 (0.59,1.99)	2.68*** (1.58,4.57)	1.97* (1.10,3.53)	1.48 (0.79,2.77)	3.15*** (1.68,5.91)
ADHD adjusted for DCD	1.63 (0.92,2.90)	2.26** (1.29,3.97)	1.10 (0.60,2.01)	2.64*** (1.56,4.49)	1.96* (1.10,3.51)	1.57 (0.83,2.98)	3.13*** (1.67,5.85)
ADHD adjusted for ASD, LD, TD and DCD	1.41 (0.76,2.62)	2.39** (1.34,4.27)	1.03 (0.55,1.92)	2.87*** (1.64,5.02)	2.35** (1.29,4.29)	1.59 (0.82,3.09)	3.31** (1.68,6.51)
ADHD adjusted for ODD	1.51 (0.86,2.67)	2.13* (1.20,3.80)	1.06 (0.57,1.98)	2.51** (1.47,4.29)	1.90* (1.06,3.40)	1.48 (0.79,2.78)	3.01** (1.59,5.67)
ADHD adjusted for CD	1.59 (0.90,2.81)	2.26** (1.28,3.99)	1.08 (0.59,1.98)	2.65*** (1.57,4.50)	1.95* (1.09,3.49)	1.47 (0.79,2.75)	3.13*** (1.67,5.88)
ADHD adjusted for OCD	1.67 (0.94,2.96)	2.25** (1.28,3.94)	1.11 (0.60,2.03)	2.68*** (1.58,4.54)	1.95* (1.09,3.48)	1.46 (0.79,2.73)	3.22*** (1.71,6.09)
ADHD adjusted for ED	1.61 (0.91,2.84)	2.30** (1.31,4.04)	1.08 (0.59,1.98)	2.69*** (1.58,4.57)	1.96* (1.10,3.51)	1.46 (0.78,2.74)	3.34*** (1.75,6.35)
ADHD adjusted for ASD, LD, TD, DCD, ODD, CD, OCD, and ED	1.48 (0.78,2.82)	2.26** (1.26,4.04)	1.03 (0.54,1.95)	2.87*** (1.61,5.12)	2.22* (1.20,4.10)	1.55 (0.79,3.01)	3.64** (1.76,7.55)
ADHD adjusted for ASD, LD, TD, DCD, ODD, CD, OCD, ED, and Parental education^A^	1.75 (0.85,3.63)	1.50 (0.75,3.01)	1.16 (0.55,2.42)	2.16* (1.14,4.09)	1.76 (0.85,3.64)	1.44 (0.64,3.25)	3.25* (1.33,7.94)

N = 293 (missing = 7), ADHD-cases compared to all other cases and controls (screen negative cotwins are not included).

Exponentiated coefficients; 95% confidence intervals in brackets

* p<0.05

** p<0.01

*** p<0.001

^A^ Parental education = Educational level of parents (mother and father)

## Discussion

The aim of the present study was to examine to what extent signs of NDPs at ages 9 or 12 years of age is associated with negative psychosocial outcome in a range of areas at age 15, while at the same time controlling for comorbidity. Our findings indicate that negative psychosocial outcomes were common in adolescence for all childhood proxy diagnoses; both for NDPs and other conditions. A further analysis of outcomes in relation to childhood ADHD symptoms showed an increased risk of negative psychosocial outcomes during adolescence, regardless of other co-existing symptoms. However, it was common that ADHD co-existed with other symptoms, and that risk levels for different outcomes changed when comorbidity was taken into account. When considering comorbidity as well as parental education level, early ADHD-symptoms still stood out as a contributing factor to both antisocial development and lowered psychosocial functioning.

### Outcome in relation to same age peers

On a group level, risk behaviors were more common in our study sample than among general Swedish 15-year olds. According to the School survey, an annual survey on criminality and drug use among Swedish 15-year olds based on self-report data, it is quite common that youth engage in illegal behavior, but the reported levels of such problems were higher in the studied cohort. For example, over one third (33.6%) of our respondents had committed at least one criminal act, compared to 20% in the national sample [77], and one third (29.5%) had committed a violent act compared to the 10% national level. It should also be noted that even though self-rate levels were higher in our study cohort than in population-based samples of the same age, parents generally reported even higher problem levels than did the participating adolescents.

Overall, there was a trend towards a higher problem load among all those who had been screen-positive for a diagnosis at ages 9 or 12 years (NDP or other) compared with those who had been screen-negative co-twins or controls, in that those who had been screen-positive reported more psychosocial problems (including antisocial behaviors) and were more often judged by assessing psychologist and child psychiatrist to have impaired functioning. Thus, not only early ADHD signs but also symptoms of other NDPs and/or other mental health and behavior problems were indicative of a more problematic adolescence. Not surprisingly, those who had been screen positive for ASD reported higher levels of peer problems, and over half were judged to have functional impairment. Given that the A-TAC, which was used for screening, has shown good to excellent psychometric properties for predicting later clinical diagnoses [52], this finding is of significant clinical relevance and stresses the importance of comprehensive assessments including symptoms other than those related to ADHD.

### Outcomes of ADHD in relation to other NDPs

Those who had been screen positive for ADHD reported the highest levels of externalizing and school problems at age 15 compared to the other groups, except for those who had been screen-positive for ODD/CD, which is to be expected. Many studies have reported similar findings of negative long-term consequences of ADHD [20, 78–85], but fewer studies have explored outcomes in relation to the entire spectrum of neurodevelopmental problems. When adjusting for all other diagnoses, including diagnoses other than NDPs, the doubled or tripled odds for ADHD in relation to several of the psychosocial outcomes remained. Previous studies have focused on the connection between ADHD and externalizing disorders such as oppositional defiant or conduct disorder [86–88] and it is well known that ADHD frequently co-occurs with externalizing disorders, and that there is a considerable overlap between ADHD and oppositional defiant or conduct disorder. However, the interplay between these conditions is complicated, and the view that ADHD is a stable condition that precedes the onset of externalizing behaviors has been challenged [88]. However, since our study sample was young, only a few were screen positive for diagnoses such as conduct disorder, which could have underestimated the impact of early externalizing behaviors.

### Socioeconomic factors and ADHD

Previous studies have also focused on the impact of socioeconomic factors on both the prevalence of ADHD and of its outcomes [80, 89, 90]. When we added a contextual factor (parental education) to our analysis, the only negative outcomes of ADHD that remained significant were antisocial behavior and impaired social functioning. Thus, the finding that parental education impacts outcome of ADHD is in line with other studies indicating that ADHD and socioeconomic factors are interconnected. In a population based study of over 800 000 Swedish individuals, Larsson et al. found a dose-dependent association with lower family income and the risk of having a child diagnosed with ADHD [91]. Currie and Stabile [80] applied the concept of human capital accumulation to study the effects of ADHD, and concluded that even though a given level of ADHD symptoms had similar effects on the academic test scores of rich and poor children, the richer children were less likely to repeat a grade. These findings were corroborated in a study by Fletcher and Wolfe [92]. In a follow-up of ADHD youth, Fried concluded that even though ADHD was the strongest predictor for academic underachievement, social class and IQ were also significant predictors of high school dropout or repeated grade [89].

### Comorbidity between NDPs

It has been suggested that single NDP diagnoses are rare and should be regarded as exceptions rather than the rule [1, 42, 93]. In the DOGSS cohort, comorbidity was common, as were negative psychosocial outcomes during adolescence. The findings clearly point out ADHD as the single most important condition for antisocial behavior during adolescence. However, the results also underscore the need for comprehensive assessment of the entire neurodevelopmental spectrum and the need to take social and family factors into consideration when planning treatment. It is likely that an individual with a combination of ADHD and ASD will need a different treatment approach and support than someone with ADHD combined with e.g. TD or LD. Therefore, assessment in early childhood focusing on a wider neurodevelopmental spectrum seems highly relevant. On the same note, a wider risk assessment perspective during adolescence is important, given the higher levels of various negative outcomes reported among the 15 year olds in this study.

## Conclusions

Signs of NDPs as well as other psychiatric diagnoses at ages 9 or 12 years are associated with a more problematic adolescence. However, despite the presence of comorbidity, early ADHD symptoms stand out as the most important risk factor for later antisocial development and impaired daily functioning.

### Strengths

The strengths of the present study lie in the whole-population-based cohort of screen-positive children, their screen-negative siblings, and controls, including the wide assessments of psychosocial outcomes. We have assessed a broad spectrum of NDPs simultaneously at baseline, and assessed a wide range of psychosocial outcomes at follow-up. These psychosocial outcomes were chosen to reflect key aspects of adolescent development and considered to be crucial for the further development into adulthood.

### Limitations

The outcome measures were based mainly on self and parent reports. The time period between the A-TAC parental assessment and the follow-up measuring the psychosocial outcomes was between three and six years. This time period in itself and the complex psychosocial problems associated with puberty may interfere with interpreting the results. There were 38 twin pairs where both twins were NDP screen-positive (23 pairs of boys and 15 pairs of girls). These twin pairs share environmental factors, which could affect our results. The effects of this have been taken care of through a robust sandwich estimator which was used to adjust for the correlated data when calculating the confidence intervals. As mentioned earlier, an analysis of the attrition group showed that there were some differences between the groups regarding sex distribution, ADHD symptoms and parental education levels. The attrition group had fewer girls, more boys, more ADHD screen-positive cases and parental education levels were more concentrated to secondary school level (Table 1). These differences suggest that the attrition group might have had a heavier problem load than the study cohort, indicating that the findings regarding psychosocial problems associated with ADHD symptoms in the study group may still be valid.

### Clinical implications

Our results show that early detection and assessment is of paramount importance in children with NDPs, and that treatment and support plans need to be individualized. This includes also assessing and treating co-existing problems, such as other NDPs, and psychosocial problems such as antisocial behavior, substance abuse and peer problems. Our results underscore that children with ADHD have a high risk of becoming marginalized in society and that they may suffer serious consequences for their education, psychosocial health and future occupational life. A wider risk assessment perspective during adolescence is important, given the higher levels of various negative outcomes for the 15-year olds in this study.

### Future research

Further studies are needed to explore how gender may modify the associations between ADHD and the psychosocial outcomes included in the present study. It would also be of interest to further explore if ADHD symptoms on a subthreshold level contribute to impairments and psychosocial problems in daily life.
